# Resolving Inflammation in CKD: The Potential of SPMs and Omega-3 Derivatives as Biomarkers and Therapeutics

**DOI:** 10.3390/biomedicines14030619

**Published:** 2026-03-10

**Authors:** Beata Franczyk, Wiktoria Lisińska, Katarzyna Hossa, Kinga Katańska, Anna Wieczorek, Aleksandra Prusak, Zuzanna Biegała, Jacek Rysz, Ewelina Młynarska

**Affiliations:** 1 Department of Nephrocardiology, Medical University of Lodz, 90-419 Lodz, Poland; 2 Department of Nephrology, Hypertension and Internal Medicine, Medical University of Lodz, 90-549 Lodz, Poland

**Keywords:** lipidomic biomarkers, lipoxins, resolvins, maresins, renal fibrosis

## Abstract

Chronic kidney disease (CKD) affects more than 10% of the population and is associated with a persistent, low-grade inflammatory state that accelerates tubulointerstitial fibrosis, worsens prognosis, and increases cardiovascular risk. This review aims to synthesize current knowledge on specialized pro-resolving mediators (SPMs) in the context of CKD pathophysiology, biomarkers, and therapeutic potential. We discuss key anti-inflammatory and pro-resolving mechanisms of SPMs that translate into nephroprotective and antifibrotic effects in experimental kidney models. The review summarizes data on EPA/DHA supplementation, including its impact on lipid profiles, inflammatory biomarkers (CRP, IL-6, TNF-α), and oxidative stress in patients with CKD. We also highlight contemporary analytical methods for biomarker assessment (LC-MS/MS, UHPLC-HRMS) and their potential for monitoring inflammatory activity across its phases (initiation, attenuation, resolution), CKD progression, and responses to ω-3/SPM-based interventions. Finally, we discuss the therapeutic potential of SPMs, as well as safety considerations and pharmacological interactions. In conclusion, SPMs and ω-3-derived mediators represent promising research and clinical targets as markers and modulators of inflammation in CKD, but require further validation in well-designed prospective studies.

## 1. Introduction

Chronic kidney disease (CKD) is a progressive disorder impacting over 10% of the global population, which translates to more than 800 million people worldwide. It has become a major global cause of death and is among the few non-communicable diseases that have shown a rising mortality rate over the past two decades [1].

CKD is primarily caused by conditions such as hypertension, diabetes, immune-mediated disorders, glomerulonephritis, tubulointerstitial diseases, and hereditary kidney disorders [2].

It is currently defined, according to international guidelines, as a persistent reduction in kidney function, indicated by an estimated glomerular filtration rate (eGFR) below 60 mL/min/1.73 m^2^, the presence of markers of kidney damage, or both, lasting for a minimum of three months [3].

Chronic, low-grade inflammation has been identified as a key feature of CKD, contributing significantly to its pathophysiology and partially accounting for the increased mortality observed in affected patients [4]. Studies indicate that C-reactive protein (CRP) levels are increased in over 50% of CKD patients starting from stage 3, with even greater prevalence observed in those with end-stage renal disease (ESRD) [5]. CKD establishes conditions that promote an elevated inflammatory state, including uremia, oxidative stress, infections, dyslipidemias, malnutrition, fluid overload, dialysis-related factors, periodontal disease, and impaired removal of inflammatory mediators [6]. This persistent, low-grade inflammation intensifies as renal function declines [7]. As a progressively important and modifiable risk factor, inflammation plays a central role in guiding effective therapeutic approaches to prevent the onset and progression of CKD in clinical practice [8].

Diabetic Kidney Disease (DKD), the leading cause of CKD worldwide, exemplifies the central role of chronic inflammation and oxidative stress in driving renal injury. Persistent hyperglycemia activates pro-inflammatory and pro-fibrotic signaling pathways, including NF-κB and NLRP3 inflammasome activation, leading to tubular damage, glomerulosclerosis and interstitial fibrosis [9,10,11]. These mechanisms overlap with inflammatory pathways observed across different CKD etiologies, underscoring the relevance of resolution-targeted therapeutic strategies.

Management of CKD primarily aims to slow disease progression to ESRD and to reduce the risk of associated complications, including cardiovascular disease, hypertension, and type 2 diabetes. However, many of the pharmacological treatments currently used in CKD carry the risk of significant adverse effects. Approaches that actively promote the resolution of inflammation represent a promising strategy for improving CKD outcomes [2]. Resolution of inflammation is a tightly regulated process involving reduced leukocyte infiltration, neutrophil apoptosis, and macrophage-mediated clearance of apoptotic cells and debris, without systemic immunosuppression. This coordinated activity reorganizes the inflammatory exudate and restores tissue homeostasis [12,13].

Lipid mediators derived from ω-3 and ω-6 polyunsaturated fatty acids have been suggested to contribute to the resolution of inflammation, providing a rationale for exploring specialized pro-resolving mediators (SPMs) as potential therapeutic targets in CKD [2]. SPMs contribute to regulating the inflammatory response by limiting neutrophil recruitment to sites of tissue injury, supporting non-inflammatory monocyte activity, and facilitating the clearance of pathogens and cellular debris [14]. These properties make SPMs a promising area of research in CKD, both as potential biomarkers of inflammation and as targets for novel therapeutic strategies.

This review aims to provide an overview of SPMs and ω-3 fatty acids derivatives in CKD, highlighting their potential as biomarkers of inflammation, modulators of disease progression, and therapeutic agents, with particular emphasis on their relevance across different stages of CKD and translational implications for clinical practice.

## 2. Pathophysiology of Inflammation in CKD

CKD is a progressive condition characterized not only by the gradual decline in renal function, but also by the presence of persistent, low-grade systemic inflammation. This chronic inflammatory state plays a pivotal role in accelerating kidney damage and significantly contributes to the development of cardiovascular and other systemic complications. Increasing evidence suggests that inflammation is not merely a consequence, but also a driver of CKD progression and poor prognosis, particularly in patients with advanced disease [15,16,17].

Importantly, chronic inflammation, oxidative stress, and metabolic disturbances form a self-perpetuating pathogenic network in CKD, in which each process amplifies the others and collectively accelerates renal injury and fibrosis.

The primary causes of renal injury in CKD incorporate a diverse range of mechanisms, including immune-mediated responses, tissue hypoxia and ischemia, exposure to exogenous nephrotoxic agents, accumulation of endogenous substances, and inherited genetic abnormalities. Regardless of the initial cause, CKD typically converges on common pathological endpoints, glomerulosclerosis and tubulointerstitial fibrosis, which ultimately contribute to the progressive loss of renal function [18].

### 2.1. Immune Dysregulation and Inflammatory Signaling in CKD

Although the initial causes of renal injury may vary, a unifying feature of CKD is the activation of innate and adaptive immune pathways that sustain low-grade inflammation and drive fibrotic remodeling. Healthy kidneys contribute significantly to immune system balance by filtering and removing circulating proinflammatory cytokines and pathogen-associated molecular patterns (PAMPs). In CKD, the progressive decline in renal function impairs this clearance mechanism, leading to the accumulation of inflammatory mediators and promoting sustained systemic immune activation and low-grade inflammation [19]. In response to injury or cellular stress, damaged renal cells release danger-associated molecular patterns (DAMPs) [20]. Both PAMPs and DAMPs are detected by cytoplasmic pattern recognition receptors (PRRs), such as toll-like receptors (TLRs), which trigger the assembly of the inflammasome-a multiprotein complex that plays a key role in innate immune responses [21]. The most extensively studied inflammasome in CKD is NLRP3 [22]. It is formed by the NLRP3 sensor protein, the adaptor ASC and caspase 1, and becomes activated by cellular stress signals. Once activated, caspase 1 initiates the processing of proinflammatory cytokines and promotes inflammatory cell death [23]. TLRs are mainly expressed on immune cells such as macrophages, T cells, B cells, dendritic cells and natural killer (NK) cells [24]. In the context of CKD, TLR activation contributes significantly to renal inflammation and fibrosis by promoting injury to resident kidney cells, recruitment of bone marrow-derived immune cells and the release of proinflammatory mediators such as MCP-1, IL-6, IL-18 and IL-1β via NF-κB/NLRP3 and mitogen-activated protein kinase (MAPK) pathways [21]. These inflammatory cascades are further amplified by oxidative stress and metabolic abnormalities, reinforcing immune activation and sustaining chronic inflammation.

Macrophages are key of both injury and repair processes in the kidney. Two functional extremes-M1 and M2-represents opposite sides of the macrophage activation spectrum. M1 macrophages are proinflammatory and produce cytokines such as TNF-α, IL-1β and IL-6 [25], along with reactive oxygen species (ROS), thereby amplifying acute inflammation and promoting tissue injury. In contrast, M2 macrophages are generally involved in resolution of inflammation and tissue remodeling. They secrete anti-inflammatory mediators such as IL-10 and insulin-like growth factor-1 (IGF-1), facilitating repair processes [26]. Beyond orchestrating inflammatory responses, macrophages also play a direct role in fibrogenesis by producing inflammation-associated fibrotic cytokines such as interleukin-13 (IL-13) and transforming growth factor β (TGF-β) and promoting extracellular matrix deposition [27,28].

Dendritic cells (DCs) are also present in the kidney, where they act as antigen-presenting cells linking innate and adaptive immunity [29]. They promote renal failure progression through recognition of glomerular antigens [30]. By presenting antigens to infiltrating T cells, DCs stimulate the release of proinflammatory cytokines and recruitment of additional immune cells, contributing to the tubulointerstitial mononuclear infiltrate, commonly observed in CKD [31].

### 2.2. Oxidative Stress and Mitochondrial Dysfunction

Alongside immune cells activation and cytokine release, oxidative stress emerges as a critical contributor to chronic inflammation and aggravated kidney damage in CKD. The progression of CKD is linked to a gradual elevation in oxidative stress (OS) [32]. ROS are byproducts of mitochondrial oxidative phosphorylation and are normally neutralized by antioxidants like superoxide dismutase (SOD) and glutathione. However, when the balance between oxidants and antioxidants is disrupted, an excess of ROS and reactive nitrogen species (RNS) accumulates causing kidney damage. This imbalance is known as OS. Due to its high oxygen consumption, the kidney is particularly susceptible to oxidative stress-related injury [21]. Reduced antioxidant capacity is an important contributing factor to chronic inflammation in CKD [16]. Hypoxia and ischemia are important contributors to renal injury [18], particularly in the context of hemodynamic disturbances, where reduced oxygen delivery impairs the efficiency of mitochondrial electron transport chain, leading to excessive production of ROS. These oxidative molecules contribute to further injury of tubular epithelial cells [33]. Mitochondrial dysfunction not only amplifies oxidative stress but also acts as a critical link between inflammation and metabolic disturbances in CKD. Damaged tubules adopt a senescence-associated phenotype, releasing profibrotic factors like TGF-β1, wingless/Int-1 (Wnt) signaling pathway and sonic hedgehog. These factors stimulate fibroblast activation and proliferation, leading to increased extracellular matrix accumulation and contributing to renal interstitial fibrosis [34]. Renal injury also triggers the activation of proinflammatory M1 macrophages [25], which release increased amount of inflammatory cytokines, ROS and nitric oxide (NO), contributing to progression of renal damage [21].

### 2.3. Altered Lipid Metabolism and Lipotoxicity in CKD

While immune activation and oxidative stress are central to inflammation in CKD, the disturbances in lipid metabolism are increasingly recognized as active participants in this process. Lipid accumulation can be observed in almost all the renal parenchyma cells in response to damage [35]. Lipids not only provide a major energy source but also play essential structural and regulatory roles in maintaining physiological homeostasis. They are broadly categorized into three major types: (1) triglycerides (TG), consisting of one glycerol and three fatty acids; (2) phospholipids and (3) lipoids. Based on the saturation of the hydrocarbon chains, fatty acids are classified into saturated fatty acids (SFAs), monounsaturated fatty acids (MUFAs), and polyunsaturated fatty acids (PUFAs). Excessive intake of SFAs has been associated with various pathologies, including cancer, obesity, myocardial infarction and insulin resistance, primarily through the activation of the NLRP3 inflammasome complex. In contrast PUFAs exhibit anti-inflammatory and protective effects, counteracting many of the detrimental processes induced by SFAs [21]. CKD is associated with a unique lipidomic pattern, including higher circulating MUFAs levels and decreased PUFAs levels [36]. The dyslipidemic profile characteristic of CKD primarily arises from defective catabolism of TG-rich lipoproteins (TRLs), such as very-low-density lipoproteins (VLDL), intermediate-density lipoproteins (IDL) and chylomicrons, alongside with high-density lipoproteins (HDL) dysfunction [37]. The impaired TRL metabolism is a result of dysregulated enzyme activity, leading to delayed clearance from the bloodstream. A key factor is the elevated plasma concentration of apolipoprotein C-III (apoC-III), which inhibits both the activity of lipoprotein lipase (LPL), the enzyme responsible for hydrolyzing TRL triglycerides and the hepatic uptake of TRL remnants. Reduced renal clearance of apoC-III in CKD patients contributes to its accumulation, further impairing LPL-mediated triglyceride catabolism [38]. In parallel, dietary fatty acids, particularly PUFAs, exert regulatory effects on lipid metabolism by modulating the activity of key transcription factors, including sterol regulatory element-binding protein 1 (SREBP-1), peroxisome proliferator-activated receptor α (PPARα) and liver X receptor α (LXRα) [39,40]. Long chain ω-3 PUFAs are capable of regulating the nuclear availability of SREBP-1 by preventing the proteolytic activation of its precursor form embedded in the endoplasmic reticulum membrane. As a result, the concentration of the transcriptionally active SREBP-1 is diminished in the presence of PUFAs [39]. Unsaturated fatty acids, in contrast to their saturated counterparts, are capable of competitively interfering with LXRα activity, limiting its interaction with DNA and thereby down regulating the expression of target genes, such as SREBP-1c [41], which is the major isoform of SREBP-1 playing a central role in activating genes responsible for the synthesis of fatty acids and TG [42].

These systemic alterations in lipid handling are not limited to circulating lipoproteins but also manifest locally within renal tissue. CKD leads to changes in lipoprotein levels and structure, primarily driven by the effects of proteinuria and the toxic uremic environment. The regulation of lipid levels within cells relies on a balance between lipid synthesis, fatty acid oxidation (FAO), lipid uptake and export. Deficiencies in mitochondrial β-oxidation, the primary pathway for fatty acid utilization, leads to lipid accumulation and contributes directly to renal injury [21]. This process is known as lipotoxicity [43]. Lipotoxicity represents a critical mechanistic intersection between metabolic dysfunction, oxidative stress, and inflammation, highlighting how altered lipid handling directly contributes to tubular injury and fibrosis [21,43]. The intracellular buildup of lipids impairs mitochondrial function and promotes oxidative stress, inflammatory responses, endoplasmic reticulum (ER) stress and renal cell apoptosis. These lipotoxic effects correlate with worsening proteinuria, progressive decline in eGFR, and the development of renal fibrosis. The accumulation of free fatty acids (FFAs) in tubular epithelial cells (TECs) and podocytes leads to mitochondrial dysfunction. Damaged mitochondria produce hydrogen peroxide, which activates c-Jun N-terminal kinase (JNK) pathway and triggers caspase-3 cleavage, resulting in apoptosis of TECs. Additionally, FFA-induced apoptosis is mediated by activation of renin–angiotensin–aldosterone system (RAAS). FFAs also promote the generation of ROS, activating the inflammasome and causing DNA damage that disrupts the cell cycle and induces cellular senescence. Senescent TECs release cytokines and chemokine that recruit immune cells such as macrophages, as well as fibrotic factors that stimulate fibroblasts activation [21]. The mitochondrion is a crucial organelle where FAO occurs, and its damage is consistently linked to impaired FAO. TGF-β, a key driver of renal fibrosis, has been shown to impair FAO by down regulating the expression of PGC-1α, a gene essential for mitochondrial biogenesis, via a Smad3-dependent signaling pathway [44].

Dysregulated lipid metabolism plays a crucial role in the progression of CKD by promoting lipotoxicity, oxidative stress and inflammation. These processes contribute to mitochondrial dysfunction, renal cell death and ultimately to tubulointerstitial fibrosis and renal impairment. Elevated levels of FFAs and modified lipoproteins further exacerbate kidney damage by activating pathogenic signaling pathways [43,45,46].

Chronic inflammation, oxidative stress and dysregulated immune responses are central contributors to the pathogenesis and progression of CKD [6,15,47]. These interconnected processes disrupt normal renal architecture through maladaptive repair and progressive tubulointerstitial fibrosis [15,18], while also promoting the accumulation of pro inflammatory metabolites due to impaired renal clearance [6,16]. Altered lipid metabolism further exacerbates inflammation and oxidative injury via mitochondrial dysfunction, lipotoxicity and activation of immune pathways [15,21,47]. Together, these mechanisms not only accelerate renal decline but also increase the risk of cardiovascular disease, partly through systemic metabolic disturbances and persistent activation of the renin–angiotensin–aldosterone system (RAAS) [29,48].

**Figure 1:**
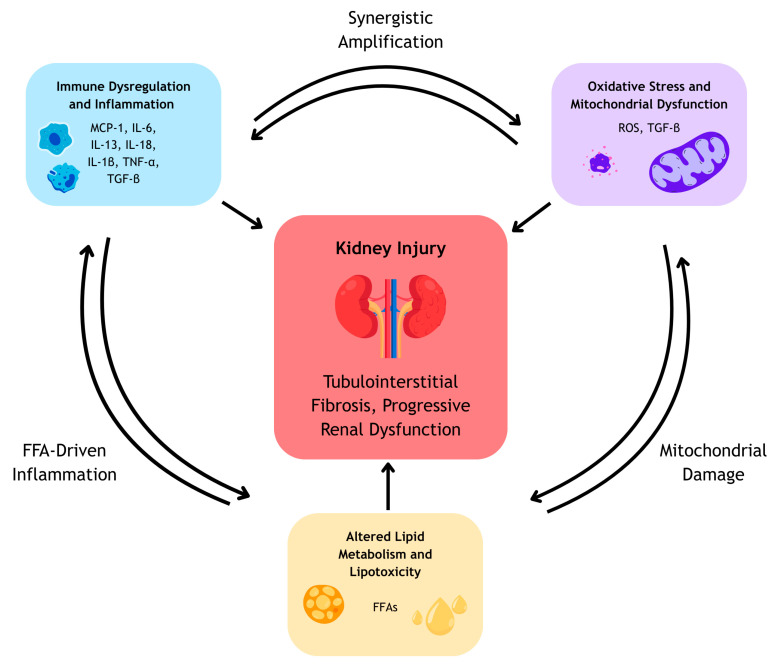
Vicious cycle of CKD progression. Abbreviations: FFAs—Free Fatty Acids; MCP-1—Monocyte Chemoattractant Protein-1; IL-6—Interleukin-6; IL-13—Interleukin-13; IL-18—Interleukin-18; IL-1β—Interleukin-1β; TNF-α—Tumor Necrosis Factor-α; TGF-β—Transforming Growth Factor-β; ROS—Reactive Oxygen Species.

In summary, CKD progression is driven by a tightly interconnected network of immune dysregulation, oxidative stress, and altered lipid metabolism. Immune activation promotes oxidative injury and metabolic dysfunction, while mitochondrial damage and lipotoxicity further amplify inflammation and fibrogenic signaling. Together, these self-reinforcing mechanisms lead to maladaptive repair, tubulointerstitial fibrosis, and progressive loss of renal function, forming the vicious cycle of CKD progression illustrated in Figure 1 [15,21,43,47].

## 3. Specialized Pro-Resolving Mediators (SPMs)

### 3.1. Definition and Classification of SPMs

SPMs are endogenous lipids mediators derived from PUFAs. They are generated in tissues during a course of anti-inflammatory response and are considered immuno-resolvents-mediators that promote the resolution of inflammation without causing immunosuppression [49,50]. A hallmark of the inflammatory response is a “class switch” from pro-inflammatory to anti-inflammatory/pro-resolving lipid mediator production. Thus, cells that initially synthesize pro-inflammatory eicosanoids later begin to produce SPMs (which has anti-inflammatory effects) from omega-3 fatty acids, such as arachidonic acid (AA, ω-6), eicosapentaenoic acid (EPA, ω-3), and docosahexaenoic acid (DHA, ω-3) [51]. Their formation from PUFSs is catalyzed by enzymes such as lipoxygenases (LOX), acylated cyclooxygenase-2 (COX-2), and monooxygenase CYP. Based on chemical structure, biosynthetic pathways, and precursor PUFAs, SPMs are grouped into five major classes: lipoxins (LX), E-series resolvins (RvE), D-series resolvins (RvD), protectins (PD), and maresins (MaR) [52].

SPMs, including D-series resolvins (RvD1, RvD2), E-series resolvins (RvE1, RvE2), protectins (PD1, PDX), and maresins (MaR1, MaR2), are biosynthesized from omega-3 fatty acids: eicosapentaenoic acid (EPA) and docosahexaenoic acid (DHA) through sequential enzymatic reactions mediated by 15-lipoxygenase (15-LOX) and 5-lipoxygenase (5-LOX) [53]. The formation of these lipid mediators involves stereospecific oxygenation of polyunsaturated fatty acids, producing hydroperoxy intermediates that are subsequently converted into bioactive SPMs. These compounds function as potent endogenous regulators of the resolution phase of inflammation, a process long considered passive but now recognized as highly orchestrated and active [54]. Mechanistically, SPMs terminate inflammation by multiple coordinated actions. They suppress neutrophil chemotaxis and transmigration into inflamed tissues, thereby preventing further tissue injury. They promote macrophage efferocytosis, the engulfment and clearance of apoptotic cells and cellular debris, which facilitates tissue healing and limits the release of proinflammatory cytokines [55]. In addition, SPMs stabilize endothelial barrier function, reduce oxidative stress, and restore vascular homeostasis through modulation of NO and ROS signaling. The D-series resolvins (e.g., RvD1 and RvD2) and maresins (e.g., MaR1) have been shown to upregulate anti-inflammatory transcription factors such as Nrf2 and PPAR-γ, while concurrently suppressing NF-κB-driven cytokine production.

### 3.2. Biosynthesis of SPMs Classes

LX (LXA_4_, LXB_4_ and their epimers 15-epi-LXA_4_/LXB_4_) are derived AA (ω-6) through enzymatic conversion involving 5-LOX and 15-LOX, primarily during intercellular interactions between neutrophils and other cell types such as epithelial cells or platelets [56].

In the classical (neutrophil–epithelial) pathway, 5-LOX in neutrophils converts AA to leukotriene A_4_ (LTA_4_), which is subsequently metabolized by 15-LOX in epithelial cells or macrophages to form LXA_4_ and LXB_4_ [57]. In the neutrophil–platelet pathway, LTA_4_ generated in neutrophils is further transformed by 15-LOX in platelets in transcellular biosynthesis (process that requires close physical proximity between neutrophils and platelets [57,58].

An additional aspirin-triggered pathway has also been described, activated, for example, in endothelial cells. Aspirin acetylates cyclooxygenase-2 (COX-2), which then produces 15R-hydroxyeicosatetraenoic acid (15R-HETE) instead of prostaglandins. In neutrophils, 15R-HETE is subsequently converted by 5-LOX to the LX epimers 15-epi-LXA_4_ and 15-epi-LXB_4_, collectively known as aspirin-triggered lipoxins (ATLs) [58,59,60].

Resolvins are synthesized from omega-3 fatty acids: eicosapentaenoic acid (EPA) gives rise to RvE1 and RvE2, while docosahexaenoic acid (DHA) serves as the precursor for RvD1-RvD6 [61]. Additionally, under the influence of COX-2, particularly in the presence of aspirin, stereoisomers known as aspirin-triggered resolvins (AT-RvD1) are generated [62,63]. Biosynthesis of RvE begins with formation of 18-HEPE from unesterified EPA by COX-2 and p450, that is next converted by leukocyte 5-LOX to RvE. DHA is transformed by 15-LOX to 17S-HDHA that is converted for RvD by 5-LOX [64,65].

Protectins are also derived from DHA through oxidation catalyzed by 15-lipoxygenase-1 (15-LOX-1), forming 17S-HDHA. This intermediate undergoes epoxidation and subsequent hydrolysis to yield the active form, protectin D1 (PD1) [65,66]. In the presence of aspirin, COX-2 generates the 17R-PD1 epimer, referred to as aspirin-triggered protectin (AT-PD1) [67].

Maresins (MaR1, MaR2) are DHA-derived lipid mediators synthesized primarily in macrophages. Their biosynthesis begins with 12-lipoxygenase (12-LOX) mediated oxidation of DHA to 14S-hydroperoxy-DHA (14S-HpDHA), which is then converted via an intramolecular reaction into 13S,14S-epoxy-maresin (epoxy-DHA). This intermediate can either be hydrolyzed to yield 7R,14S-diHDHA (MaR1) or metabolized by soluble epoxide hydrolase (sEH) to form MaR2 (13R,14S-diHDHA) [68,69].

**Figure 2:**
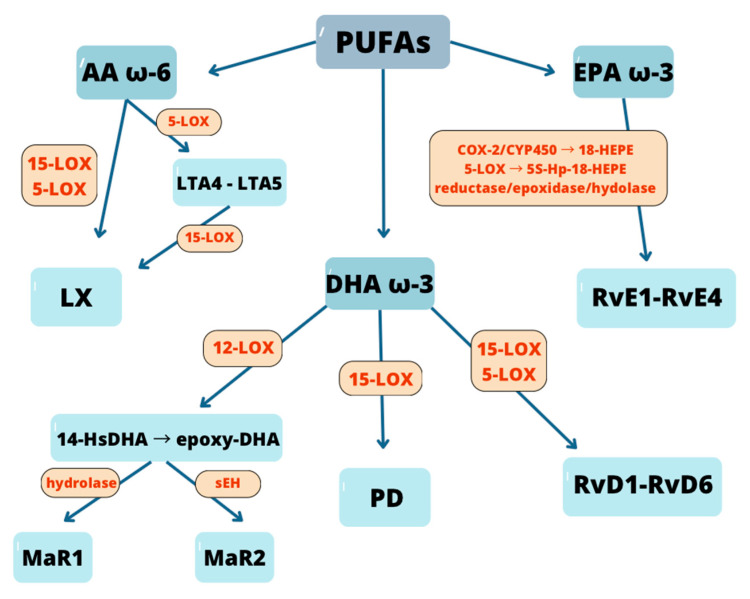
Schematic representation of SPM biosynthesis and their key enzymes. Abbreviations: PUFAs—Polyunsaturated fatty acids, AA—Arachidonic acid, EPA—Eicosapentaenoic acid, DHA—Docosahexaenoic acid, 5-LOX—5-Lipoxygenase, 12-LOX—12-Lipoxygenase, 15-LOX—15-Lipoxygenase, LTA4/5—Leukotriene A4/5, LX—Lipoxins, COX-2—Cyclooxygenase-2, CYP450—Cytochrome P450 enzymes, 18-HEPE—18-Hydroxyeicosapentaenoic acid, 5S-Hp-18-HEPE—5S-Hydroperoxy-18-hydroxyeicosapentaenoic acid, RvE—Resolvin E series, RvD—Resolvin D series, 14-HsDHA—14-Hydroxy-docosahexaenoic acid, sEH—Soluble epoxide hydrolase, MaR—Maresin, PD—Protectins.

The biosynthetic pathways of SPMs classes are summarized in Figure 2.

### 3.3. Anti-Inflammatory Mechanisms of SPMs

Lipoxins (LX) actively terminate inflammation through multiple mechanisms: they inhibit neutrophil chemotaxis and adhesion to the endothelium, promote neutrophil apoptosis, and enhance efferocytosis by macrophages. They reprogram macrophages toward an anti-inflammatory phenotype (M2), thereby increasing IL-10 production and supporting tissue repair [70,71,72,73,74]. At the receptor level, by competing for ALX/FPR2, LX suppress NF-κB activation and lower TNF-α and IL-8 levels, attenuating pro-inflammatory signaling [75,76]. In addition, by inhibiting the MAPK/AKT and Smad cascades and restraining the TGF-β/TGF-βR1 axis (partly via induction of miR-let-7c) they dampen pro-inflammatory responses and profibrotic processes [77,78,79]. Consistent effects have been observed in diabetic kidney disease (DKD) models, where LXA_4_ administration silenced immediate-early response genes (EGR-1) and the TNF-α/TGF-β/NF-κB axis, resulting in a systemic reduction in inflammatory mediators and stabilization of a pro-resolving phenotype in innate immune cells, alongside decreased albuminuria, reduced mesangial expansion, antifibrotic actions, and partial reversal of established DKD features [80].

The mechanism of action of resolvins involves multi-directional termination of inflammation. At the molecular level, resolvins signal through specific G protein-coupled receptors: RvE1 activates ChemR23 and antagonizes leukotriene B_4_ receptor 1 (BLT1), thereby limiting leukocyte migration and inflammatory signaling [81]. RvD1 signals via ALX/FPR2 and GPR32 [82], whereas RvD2 signals via GPR18 [83]. Activation of these receptors suppresses the transcription of pro-inflammatory genes through inhibition of NF-κB pathways [84,85]. At the cellular level, resolvins inhibit neutrophil chemotaxis to inflammatory sites, promote efferocytosis, and reprogram macrophages toward an M2 phenotype, resulting in decreased TNF-α, IL-1β, and IL-6 with concomitant increases in IL-10 and TGF-β [5]. Additionally, studies have shown that RvD1 stabilizes mitochondrial homeostasis and reduces oxidative stress, reinforcing a pro-resolving phenotype in innate immune cells [84]. In models of CKD, resolvins also exhibit anti-fibrotic actions: in adriamycin (ADR)-induced nephropathy, RvD1 treatment attenuated proteinuria, glomerulosclerosis, and tubulointerstitial fibrosis, shifted macrophages from an M1 to M2 phenotype, and exerted renoprotective effects on podocytes [86]. In unilateral ureteral obstruction (UUO), RvE1, via ChemR23, blocks platelet-derived growth factor-BB (PDGF-BB) signaling in fibroblasts, leading to reduced collagen deposition and α-SMA expression; this study confirms a direct anti-proliferative effect of RvE1 on fibroblasts [87].

MaR, especially MaR1, exhibit anti-inflammatory and pro-regenerative actions: they inhibit neutrophil migration, enhance phagocytosis and efferocytosis, and reprogram macrophages toward an M2 phenotype, while simultaneously lowering TNF-α, IL-1β, IL-6, and IFN-γ [88,89,90]. MaR1 acts primarily via the LGR6 GPCR [91] and the nuclear receptor RORα (which promotes 12-LOX expression and supports MaR1 biosynthesis through positive feedback) [92]. By limiting inflammation and oxidative stress, they exert nephroprotective and anti-fibrotic effects and support regeneration of injured nephrons. In the ischemia/reperfusion (I/R) model, MaR1 suppresses TLR4/MAPK/NF-κB pathways, activates Nrf2, decreases TNF-α/IL-6, and increases IL-10, thereby reinforcing the M2 phenotype [90]. In diabetic nephropathy, it normalizes glycemia, reduces albuminuria and cytokine expression, and by LGR6 activation → ↑cAMP/↑SOD2 reduces ROS and inflammation [93].

The key receptor for PD1 is GPR37, which enhances phagocytosis and supports the resolution of inflammation [94,95,96]. Protectins exhibit anti-inflammatory actions: even a single PD1 dose (~1 ng) reduces neutrophil influx to inflammatory sites by approximately 40% [67], while concurrently augmenting efferocytosis and macrophage polarization toward the M2 phenotype via PPARγ activation, and lowering pro-inflammatory cytokines (TNF-α, IL-6, MCP-1) with a concomitant increase in IL-10 [97]. Additionally, PD1 inhibits TLR-dependent macrophage activation, thereby suppressing pro-inflammatory cascades in the kidney [98]. Protectins also display an antioxidant component by upregulating SOD2 and downregulating NOX2, which reduces ROS generation and the persistence of inflammatory responses in the urinary system [99].

**Figure 3:**
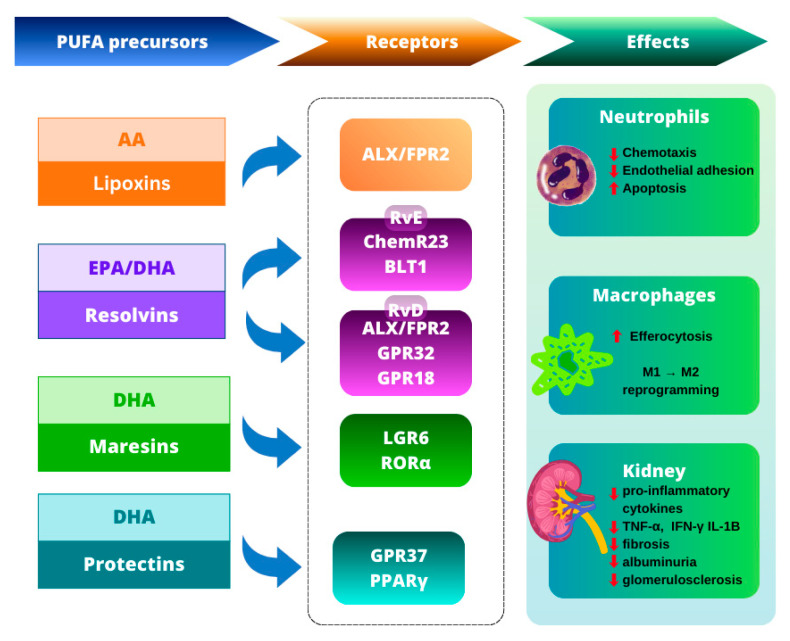
Schematic overview of PUFA-derived SPMs, their receptors, and downstream immuno-renal effects. Abbreviations: PUFA—Polyunsaturated fatty acids, AA—Arachidonic acid, EPA—Eicosapentaenoic acid, DHA—Docosahexaenoic acid, RvE—Resolvin E series, BLT1—Leukotriene B4 receptor 1, RvD—Resolvin D series, GPR32/18—G protein-coupled receptor 32/18, LGR6—Leucine-rich repeat-containing G protein-coupled receptor 6, RORα—Retinoic acid receptor-related orphan receptor alpha, GPR37—G protein-coupled receptor 37, PPARγ—Peroxisome proliferator-activated receptor gamma, TNF-α—Tumor necrosis factor alpha, IFN-γ—Interferon gamma, IL-1β—Interleukin 1 beta.

In summary, SPMs actively terminate inflammation by inhibiting neutrophil chemotaxis and adhesion, enhancing efferocytosis, and reprogramming macrophages toward an M2 phenotype [5,70,71,72,73,74,88,89,90,97]. They act via specific GPCRs-including ALX/FPR2, ChemR23, GPR32, GPR18, and LGR6-thereby suppressing NF-κB/AP-1/TLR pathways [75,76,77,78,81,82,83,84,85,90,91,98] and reducing pro-inflammatory cytokine expression while increasing IL-10 [5,75,88,89,90,97]. In renal models, these mechanisms translate into nephroprotective and anti-fibrotic effects across acute kidney injury (AKI), I/R, sepsis, DKD, UUO, ADR, and nephrolithiasis, reinforced by antioxidant actions (↑Nrf2/SOD2, ↓NOX2/ROS) [80,86,87,90,99]. A schematic overview summarizing SPMs, their receptors, and downstream immuno-renal effects is shown in Figure 3. Overall, SPMs are key pro-resolving mediators that not only blunt the acute inflammatory phase but also support tissue repair and limit long-term fibrotic remodeling in the kidney [80,86,87,90,99]. A summary of the major SPM classes, their precursors, biosynthetic pathways, receptors, and representative renal effects is presented in Table 1.

## 4. Omega-3 Fatty Acid Derivatives

### 4.1. Overview of Omega-3 Polyunsaturated Fatty Acids (PUFAs)

In recent years, interest in PUFAs has grown significantly due to their important roles in supporting health and reducing the risk of various diseases. This group of fatty acids includes α-linolenic acid (ALA; 18:3 ω-3), stearidonic acid (SDA; 18:4 ω-3), eicosapentaenoic acid (EPA; 20:5 ω-3), docosapentaenoic acid (DPA; 22:5 ω-3), and docosahexaenoic acid (DHA; 22:6 ω-3).

Oils containing these fatty acids-either entirely or in part-are derived mainly from specific plant sources or produced through plant modification, as well as from marine, algal, and single-cell origins. Long-chain (LC) ω-3 fatty acids such as EPA and DHA are primarily found in the body lipids of oily fish, the liver of lean fish, and the blubber of marine mammals.

Although marine organisms remain the primary source of ω-3 PUFAs, certain plant seeds also contain them. For instance, flaxseed, chia, and canola are rich in ALA, which acts as a precursor for the synthesis of long-chain PUFAs in the human body. However, the conversion rate of ALA to LC ω-3 PUFAs is very limited-typically less than 4%. Therefore, including LC ω-3 PUFAs directly in the daily diet is essential to meet the body’s physiological needs [100,101].

### 4.2. Metabolic and Regulatory Effects of Omega-3 Fatty Acids

Omega-3 fatty acids and their eicosanoid derivatives have demonstrated multiple benefits in individuals with cardiovascular disease, diabetes [102], cancer [103], and nephropathies [100]. Nevertheless, despite their potential advantageous effects, ω-3 supplementation is not yet routinely recommended for patients with CKD.

Lipid abnormalities typical of CKD include elevated triglyceride levels and reduced high-density lipoprotein cholesterol (HDL-C) [104]. Patients with CKD exhibit a characteristic fatty acid pattern marked by increased serum monounsaturated fatty acid (MUFA) levels and decreased PUFA concentrations [36].

EPA and DHA modulate the activity of transcription factors such as SREBP-1c, LXRα, and PPARα, which control fatty acid synthesis and oxidation [39,40,41,105]. EPA inhibits the proteolytic processing of the inactive SREBP-1c precursor into its active form, thereby reducing the expression of lipogenic genes [39]. Additionally, ω-3 PUFAs can act as antagonists of LXRα, impairing its DNA-binding activity and further decreasing SREBP-1c expression [40,41].

Furthermore, EPA and DHA are natural ligands for PPARα, the predominant transcription factor in oxidative tissues. Activation of PPARα enhances the expression of genes involved in fatty acid oxidation, promoting hepatic lipid catabolism [105,106]. Increased dietary intake of ω-3 PUFAs promotes the channeling of fatty acids into oxidative pathways, modulates triglyceride metabolism, and may help regulate plasma lipid levels.

### 4.3. Effects of Omega-3 Fatty Acid Supplementation on Lipid Profile in Patients with CKD

Numerous clinical studies evaluating the effects of ω-3 fatty acid (EPA and DHA) supplementation in patients with CKD and those undergoing dialysis (hemodialysis (HD) or peritoneal dialysis) have produced inconclusive results. Several clinical trials reported no significant effect of ω-3 PUFA supplementation on the serum lipid profile [101,107,108], whereas others confirmed a beneficial impact—namely, a significant reduction in triglyceride (TG) levels [109,110], sometimes accompanied by decreases in total cholesterol and increases in HDL-C [32].

### 4.4. Anti-Inflammatory and Antioxidant Effects

CKD represents a state of persistent microinflammation, with inflammatory processes being a prominent feature in both predialysis and dialysis patients [111]. Omega-3 PUFAs modulate inflammation primarily through interactions with the arachidonic acid (AA) cascade.

Their intake elevates EPA levels, which act as a competitive inhibitor of cyclooxygenase, leading to reduced synthesis of 2-series prostaglandins, thromboxanes, and prostacyclins, as well as 4-series leukotrienes. Concurrently, ω-3 PUFAs promote the formation of 3- and 5-series prostanoids that exhibit lower biological activity.

Docosahexaenoic acid (DHA), another ω-3 PUFA, suppresses AA metabolism and platelet aggregation by decreasing the affinity of the platelet TXA_2_/PGH_2_ receptor [112]. In experimental models, ω-3 PUFA supplementation markedly suppressed oxidative and inflammatory pathways by downregulating the expression of NOX4, gp91phox, p47phox, p22phox, MCP-1, NF-κB, and COX-2 in a 5/6 nephrectomy rat model, contributing to a significant reduction in renal fibrosis [113].

### 4.5. Effects on Inflammatory Markers and Clinical Outcomes

Recent clinical studies have reinforced the therapeutic relevance of omega-3 fatty acid derivatives in CKD, emphasizing their ability to modulate inflammation, oxidative stress, and metabolic dysregulation beyond their nutritional role. In the dialysis population, supplementation with PUFAs has been associated with a reduction in inflammatory biomarkers, including CRP and tumor necrosis factor-α (TNF-α) [114]. Furthermore, a prospective, randomized, double-blind clinical trial including patients with CKD undergoing HD demonstrated that a 12-week ω-3 PUFA supplementation regimen significantly decreased serum levels of inflammatory mediators such as interleukin-6 (IL-6), CRP, and TNF-α [115]. Also pilot study evaluated that raising blood ω-3 PUFA levels by fish oil supplementation significantly reduced CRP levels in HD patients [116]. In a randomized, double-blind, placebo-controlled trial involving patients with end-stage renal disease receiving maintenance hemodialysis, six-month supplementation with 1 g/day of omega-3 syrup containing eicosapentaenoic acid (EPA, 825 mg) and docosahexaenoic acid (DHA, 550 mg) markedly improved antioxidant defense and attenuated systemic inflammation. The treatment enhanced the activity of glutathione peroxidase, a key antioxidant enzyme, and lowered circulating CRP, accompanied by favourable modulation of lipid metabolism [117]. A 2024 meta-analysis encompassing thirteen randomized controlled trials further confirmed these effects, demonstrating that fish-oil supplementation (EPA + DHA) significantly reduced serum CRP (*p* = 0.01), particularly among participants with baseline CRP ≥ 5 mg/L, while changes in IL-6 and TNF-α remained inconsistent. These findings collectively indicate that omega-3 supplementation can attenuate systemic inflammation in dialysis patients, although the heterogeneity across studies underscores the need for larger, standardized clinical trials [118].

Observational data from a large 2024 population-based cohort study (UK Biobank) including more than 80,000 participants, of whom 6735 had established CKD, provided novel insights into the relationship between plasma polyunsaturated fatty acids (PUFAs) and renal outcomes. During a median 11.9-year follow-up, higher circulating PUFA levels were associated with a lower incidence of CKD in the general population, whereas among individuals with established CKD, only higher concentrations of the omega-3 fatty acid docosahexaenoic acid (DHA) correlated with a reduced risk of kidney failure requiring replacement therapy. These findings suggest that DHA may exert a specific nephroprotective effect in progressive CKD, while overall PUFA sufficiency could help maintain kidney health in the broader population [119].

Complementary mechanistic preclinical evidence indicates that another omega-3 derivative, EPA, and its downstream metabolites can attenuate renal fibrosis and prevent the transition from acute kidney injury to CKD by suppressing TGF-β1-induced fibroblast activation, thereby targeting key early processes driving CKD progression [120]. A randomized, double-blind, placebo-controlled trial found that daily supplementation with ω-3 polyunsaturated fatty acids did not affect overall mortality or the incidence of cardiovascular events in CKD patients on hemodialysis with pre-existing cardiovascular disease; however, it significantly reduced the incidence of myocardial infarction in these patients [121]. A systematic review and meta-analysis of 60 RCTs indicated that n-3 PUFA supplementation may lower the risk of cardiovascular death in hemodialysis patients, although it remains unclear whether it can prevent overall mortality or progression to end-stage kidney disease (ESKD) in individuals with CKD [122].

Collectively, these data underscore the therapeutic potential of omega-3-derived mediators as adjunctive interventions to modulate inflammation and fibrotic remodeling in CKD. Although several randomized controlled trials in patients with chronic kidney disease (CKD) have demonstrated beneficial effects of omega-3 fatty acid supplementation on inflammatory markers, the dose–response relationship remains insufficiently characterized in this population. In contrast to meta-analysis conducted in cardiovascular and metabolic populations, which report non-linear dose response in patients with cardiovascular disease, metabolic syndrome, and hypertension up to 1200 mg/day of EPA and DHA or linear dose–response associations between EPA/DHA intake and CRP reduction in dyslipidemia population, CKD-specific studies have generally evaluated single-dose interventions without formal dose-comparative analyses. Consequently, while reductions in CRP and other inflammatory parameters have been observed at doses ranging from approximately 1 to 4 g/day, current evidence in CKD does not allow definitive conclusions regarding the optimal anti-inflammatory dose. This highlights an important gap in the literature and underscores the need for future dose–response trials specifically designed for CKD populations [123]

## 5. SPMs and Omega-3 Derivatives as Biomarkers in CKD

### 5.1. Current Analytical Methods

The measurement of SPMs and omega-3 fatty acid derivatives in biological samples has become a central focus in biomarker discovery for CKD. Contemporary analytical approaches are grounded in advanced lipidomic platforms, primarily high-performance liquid chromatography coupled with tandem mass spectrometry (LC–MS/MS) and, more recently, ultra-high-performance liquid chromatography (UHPLC) combined with high-resolution mass spectrometry (HRMS). These methods enable the detection and quantification of SPMs such as resolvins (RvD, RvE series), protectins (PD1, PDX), and maresins (MaR1, MaR2) at picomolar concentrations. Quantitative analysis requires rigorous sample preparation, including lipid extraction under low-oxidative conditions, stabilization with antioxidants, and use of deuterated internal standards. Recent developments in targeted lipidomics and multiplexed metabolomic assays allow for simultaneous assessment of multiple SPM families and their precursors derived from EPA and DHA. Furthermore, immunoassays, though less specific, are under investigation for clinical feasibility, particularly enzyme-linked immunosorbent assays (ELISAs) adapted for resolvins and protectins. Integrating these analytical advances with bioinformatics-driven lipidomic profiling offers a promising route to establishing reliable SPM-based signatures associated with renal inflammation and function [124].

### 5.2. Biomarker Potential: Inflammatory Activity, CKD Progression, and Therapeutic Response

SPMs and omega-3-derived mediators hold significant promise as biomarkers reflecting distinct aspects of CKD pathophysiology as it is shown in Table 2.

#### 5.2.1. Markers of Inflammatory Activity

Persistent, low-grade inflammation constitutes a central mechanism underlying CKD progression and its systemic complications [125]. Chronic activation of innate immunity, oxidative stress, and impaired clearance of inflammatory debris contribute to endothelial injury, glomerulosclerosis, and tubulointerstitial fibrosis. While conventional inflammatory biomarkers such as CRP, IL-6, or TNF-α indicate the presence of inflammation, they fail to capture the efficacy of its resolution.

In CKD patients, several clinical and experimental studies have demonstrated an imbalance between pro-inflammatory and pro-resolving lipid mediators. Decreased circulating and urinary concentrations of RvD1, RvE1, and MaR1 have been reported in association with elevated oxidative stress markers, including malondialdehyde (MDA), F2-isoprostanes, and advanced oxidation protein products (AOPPs). This inverse relationship underscores a state of resolution deficiency, wherein the body’s ability to actively terminate inflammation is impaired despite persistent activation of pro-inflammatory pathways. Such imbalance contributes to a chronic inflammatory phenotype, endothelial dysfunction, and microvascular injury, all characteristic features of CKD progression. Moreover, emerging evidence indicates that SPM deficiency may also impair the anti-inflammatory polarization of macrophages toward the M2 phenotype, resulting in prolonged tissue damage and fibrosis. For instance, experimental models of renal ischemia–reperfusion injury have shown that exogenous administration of RvD1 or MaR1 restores M2 macrophage activity, accelerates tubular repair, and reduces interstitial fibrosis. These findings suggest that measuring SPM levels may reflect not only the inflammatory burden but also the tissue’s intrinsic reparative capacity. Consequently, profiling SPM concentrations or calculating SPM-to-cytokine ratios such as RvD1/IL-6, MaR1/TNF-α, or PD1/CRP could provide a dynamic biomarker index of inflammation resolution efficiency. Such indices would allow discrimination between active inflammation, resolving inflammation, and defective resolution, which is particularly relevant in CKD where chronic subclinical inflammation drives disease progression. This approach could enhance early detection of immune dysregulation before irreversible structural kidney damage occurs, complementing standard inflammatory panels used in clinical nephrology. Integration of SPM profiling into longitudinal patient monitoring or therapeutic trials may also help identify subpopulations with impaired resolution pathways, potentially guiding the application of omega-3 supplementation or pro-resolving mediator analogs as adjunctive therapies. Overall, SPMs represent a mechanistically grounded, quantifiable, and biologically meaningful class of biomarkers with strong translational potential in the management of CKD-related inflammation [126].

#### 5.2.2. Markers of CKD Progression

SPMs and omega-3-derived lipid mediators are increasingly recognized not only as modulators of inflammation but also as dynamic indicators of disease trajectory in CKD [127]. The progressive decline in renal function, manifested by reduced eGFR, elevated proteinuria, and histopathological evidence of glomerulosclerosis and interstitial fibrosis, is closely associated with impaired biosynthesis of specialized pro-resolving mediators. The key enzymes catalyzing SPM formation, 15-lipoxygenase (ALOX15) and 5-lipoxygenase (ALOX5), show diminished expression and activity in both renal tissue and circulating immune cells of CKD patients. This enzymatic downregulation contributes to an imbalance between pro-inflammatory eicosanoids (e.g., leukotriene B4, prostaglandin E2) and pro-resolving mediators (e.g., RvD1, MaR1, PD1), perpetuating chronic inflammation and tissue injury [128]. Experimental studies have provided strong mechanistic support for the role of SPM deficiency in renal fibrosis and structural deterioration. In murine models of unilateral ureteral obstruction (UUO) and adenine-induced nephropathy, pharmacological inhibition or genetic deletion of ALOX15 accelerates tubulointerstitial fibrosis, collagen type I and III accumulation, and upregulation of pro-fibrotic mediators such as transforming growth factor-beta 1 (TGF-β1), connective tissue growth factor (CTGF), and fibronectin [129,130]. Conversely, exogenous administration of SPMs, particularly RvD1, RvD2, or MaR1 has been shown to attenuate extracellular matrix deposition, reduce macrophage infiltration, and normalize the expression of fibrotic and oxidative stress markers. These mediators also enhance autophagic flux and promote mitochondrial homeostasis, mechanisms that may contribute to the preservation of tubular epithelial cell integrity under uremic stress [131,132]. Clinical observations mirror these experimental findings. Lower plasma and urinary concentrations of RvE1, PD1, and MaR1 have been consistently reported in patients with advanced CKD (stages 4–5) compared with early-stage disease or healthy controls. These reductions correlate with key markers of renal decline, including lower eGFR, increased albumin-to-creatinine ratio, and higher circulating levels of inflammatory cytokines such as IL-6, TNF-α, and MCP-1 [133]. Furthermore, altered SPM profiles have been associated with major CKD-related comorbidities, including vascular calcification, endothelial dysfunction, and renal anemia, conditions that reflect systemic consequences of unresolved inflammation and metabolic dysregulation. The depletion of SPMs in uremic plasma may thus signify a systemic failure in inflammation resolution, contributing to accelerated cardiorenal deterioration [134].

The concept of an SPM signature, a composite profile encompassing multiple SPM species and their biosynthetic ratios, has emerged as a promising prognostic biomarker for CKD progression. Integrating SPM concentrations into predictive models that include eGFR, albuminuria, and cardiovascular risk factors has the potential to improve early identification of high-risk patients and refine prognostic accuracy regarding time to dialysis initiation or mortality [135]. Beyond statistical associations, this approach provides mechanistic insight into the molecular underpinnings of renal decline, emphasizing the loss of endogenous pro-resolving capacity as a driver of disease evolution. Future studies should focus on validating SPM-based biomarkers in large, longitudinal CKD cohorts and exploring whether therapeutic interventions that restore SPM biosynthesis such as omega-3 supplementation or synthetic SPM analogs translate into measurable improvements in renal outcomes. Establishing standardized panels that combine lipid mediator quantification with inflammatory and fibrotic markers could pave the way toward precision medicine approaches in CKD, enabling clinicians to stratify patients by resolution potential and tailor anti-inflammatory therapies accordingly [134].

Biological roles and biomarker relevance of SPMs and omega-3 derivatives vary substantially across different stages of CKD, underscoring the importance of stage-specific interpretation for clinical application. In early CKD (stages 1–2), modest reductions in circulating or urinary SPMs often precede overt declines in eGFR or the onset of significant proteinuria [136]. At this stage, impaired SPM biosynthesis primarily reflects subtle immune dysregulation and endothelial dysfunction rather than irreversible structural injury [137]. Decreased levels of RvD1, RvE1, or MaR1 in early CKD have been associated with heightened oxidative stress and low-grade inflammation, suggesting that SPMs may serve as early warning biomarkers of defective inflammation resolution and heightened susceptibility to disease progression. In this context, SPM profiling could help identify patients who may benefit most from early preventive interventions, such as dietary omega-3 supplementation or lifestyle modification, before fibrotic remodeling becomes established [138].

In contrast, advanced CKD (stages 4–5) is characterized by a profound and systemic depletion of SPMs, driven by uremia-associated suppression of lipoxygenase activity, mitochondrial dysfunction, and chronic immune activation. At these stages, reduced SPM levels correlate more strongly with irreversible pathological features, including extensive tubulointerstitial fibrosis, vascular calcification, and heightened cardiovascular risk [139]. Consequently, in late-stage CKD, SPMs may function less as early diagnostic markers and more as indicators of disease severity, resolution failure, and therapeutic responsiveness [140]. This stage-dependent shift suggests that while SPMs hold promise as preventive and stratification biomarkers in early CKD, their greatest utility in advanced disease may lie in monitoring inflammation resolution capacity, predicting complications, and evaluating response to anti-inflammatory or pro-resolving therapies. Recognizing these stage-specific roles is essential for translating SPM measurements into clinically meaningful decision-making frameworks [141].

In humans, most evidence regarding SPMs in CKD derives from cross-sectional lipidomic analyses of plasma or urine samples, with liquid chromatography–tandem mass spectrometry (LC–MS/MS) currently representing the only validated and reliable detection platform for SPM quantification in research settings. Using this approach, several studies have reported reduced circulating or urinary levels of RvD1, RvE1, MaR1, and protectin D1 in patients with impaired renal function compared with healthy controls, with inverse associations between SPM concentrations and markers of systemic inflammation, oxidative stress, endothelial dysfunction, or albuminuria. These findings demonstrate analytical feasibility and biological association but are limited by modest cohort sizes, single time-point measurements, and heterogeneity across CKD stages [93,137,142,143].

Representative human studies of SPMs in CKD are summarized in Table 3, illustrating both the types of SPMs measured and the current translational stage of research:

From a clinical translation perspective, SPM-based biomarkers in CKD have not yet progressed beyond discovery and early validation phases. At present, no standardized clinical-grade assays, reference ranges, or threshold standards for individual SPMs have entered formal clinical validation or regulatory qualification pathways. LC–MS/MS protocols, while robust in research laboratories, remain technically demanding, costly, and insufficiently harmonized for routine clinical deployment [137].

Consequently, current evidence supports SPMs primarily as mechanistically informed indicators of impaired inflammation resolution, complementing rather than replacing established clinical measures such as eGFR and albuminuria [93]. Advancing SPMs toward clinical implementation will require large, well-phenotyped longitudinal CKD cohorts, inter-laboratory assay standardization, and regulatory-grade biomarker qualification studies to define reproducible reference intervals, validate prognostic performance, and determine their added value in clinical decision-making frameworks. Integrating SPM quantification with traditional markers of renal function and cardiovascular risk may ultimately refine patient stratification, enable early intervention, and provide mechanistic insight into the resolution failure that drives CKD progression [93,137,142,143].

#### 5.2.3. Markers of Therapeutic Response

Beyond diagnostic and prognostic value, SPMs are increasingly recognized as predictive biomarkers of therapeutic efficacy in CKD. Their circulating and urinary concentrations provide a dynamic reflection of the inflammatory resolving balance, integrating the metabolic, immune, and vascular responses to treatment [144]. Because SPM biosynthesis is enzymatically regulated and responsive to both nutritional and pharmacological modulation, these mediators can serve as sensitive indicators of biological response well before conventional markers, such as CRP or eGFR, exhibit measurable change [145].

Dietary interventions enriched in omega-3 PUFAs notably EPA and DHA have been shown to enhance endogenous SPM production through upregulation of 15-lipoxygenase (ALOX15) and 5-lipoxygenase (ALOX5) activity. In both preclinical models and CKD patients, omega-3 supplementation increases plasma and urinary concentrations of RvD1, RvE1, and MaR1, which coincide with decreased systemic inflammation, improved endothelial-dependent vasodilation, and attenuation of oxidative stress markers such as malondialdehyde (MDA) and advanced oxidation protein products (AOPPs). Such findings underscore the concept that SPM restoration reflects not merely passive anti-inflammatory suppression but active reprogramming toward immune homeostasis and tissue repair [51,138,146,147,148]. Clinical intervention studies further support this view. In randomized trials involving CKD and cardiovascular comorbidity, rising plasma RvD1 and MaR1 levels were associated with favorable lipid remodeling, reduced CRP and IL-6, and stabilization of eGFR. Similarly, pharmacological treatments known to modulate inflammatory resolution pathways such as statins, angiotensin receptor blockers (ARBs), and novel synthetic SPM mimetics (e.g., 17R-RvD1 analogs) demonstrate the capacity to upregulate pro-resolving signaling via G protein-coupled receptors (GPCRs) including ALX/FPR2 and ChemR23.

These pathways converge on the suppression of NF-κB-mediated transcription and enhancement of Nrf2 and PPAR-γ signaling, culminating in reduced oxidative damage and pro-fibrotic gene expression (TGF-β1, CTGF) [149]. Given these mechanistic underpinnings, serial monitoring of SPM levels during therapy could enable the early identification of responders and non-responders, guiding personalized treatment optimization. By integrating SPM dynamics into clinical decision-making, clinicians may tailor interventions not only based on disease stage but also on the patient’s intrinsic capacity to resolve inflammation. Moreover, SPMs represent promising mechanistic biomarkers in interventional and translational studies. Measuring changes in their profiles provides a functional link between molecular-level anti-inflammatory effects and clinically relevant outcomes such as renal function preservation, proteinuria reduction, and cardiovascular protection. Incorporating SPM quantification into future RCTs could refine endpoint interpretation, distinguish pharmacodynamic versus clinical responders, and accelerate the development of SPM-targeted therapeutic strategies in CKD management [141,150].

## 6. Therapeutic Potential of SPMs in CKD

### 6.1. Renoprotective Actions of SPMs: Experimental and Translational Evidence

CKD is increasingly understood as a state of persistent, non-resolving inflammation accompanied by oxidative stress and tubular epithelial injury, which collectively drive extracellular matrix accumulation and interstitial fibrosis, ultimately impairing renal architecture and function [151]. In view of this pathogenic profile, therapeutic interest has shifted from simple suppression of inflammatory mediators toward strategies that restore the endogenous resolution of inflammation. Within this framework, omega-3 fatty acid derivatives and SPMs have emerged as promising candidates for targeted nephroprotection in CKD [152]. Conventional treatment slows disease progression but fails to fully extinguish the underlying inflammatory drive and may be limited by adverse effects such as hyperkalemia during RAAS blockade or statin intolerance, underscoring the need for resolution-focused therapeutic approaches [2].

Among SPMs, MaR1 has emerged as a potent renoprotective mediator. A translational study integrating clinical, animal, and cellular models demonstrated that serum MaR1 concentrations were significantly lower in patients with DKD compared with both healthy controls and diabetic individuals without renal involvement. In diabetic mice, MaR1 administration markedly reduced albuminuria, tubular injury, and inflammatory cytokine expression, while enhancing antioxidant defenses via activation of the LGR6–cAMP–SOD2 pathway. In cultured renal tubular epithelial cells, MaR1 reversed high-glucose-induced oxidative injury and cytokine release in an LGR6-dependent manner [93].

Consistently, in a murine model of renal IRI, MaR1 treatment significantly improved renal function and histopathology by modulating the inflammatory response, reducing pro-inflammatory cytokines such as TNF-α and IL-6, while enhancing IL-10, a key mediator of the resolution phase of inflammation. It also decreased oxidative stress markers and upregulated antioxidant enzymes, confirming its dual anti-inflammatory and antioxidant activity [90]. Additionally, in a murine model of lipopolysaccharide (LPS)-induced septic AKI, MaR1 lowered serum creatinine and blood urea nitrogen (BUN), reduced tubular injury and inflammatory cytokine expression, and attenuated oxidative and apoptotic damage through inhibition of the NOX4/ROS/NF-κB pathway [153].

Although these experimental models differ in their underlying causes (diabetic, ischemic, and septic), they converge on overlapping pathogenic pathways characterized by chronic inflammation, oxidative stress, mitochondrial dysfunction, and apoptosis, which are fundamental drivers of CKD development and progression. The consistent renoprotective effects of MaR1 observed across these conditions demonstrate its ability to modulate key pathogenic pathways implicated in renal injury and highlight its promise as a potential therapeutic target in CKD [90,93,153].

Importantly, accumulating evidence suggests that at least part of the renoprotective effects attributed to omega-3 fatty acids in DKD may be mediated by their downstream conversion into SPMs. In DKD, defective resolution in the setting of metabolic stress and oxidative imbalance may contribute to reduced pro-resolving mediator availability, thereby sustaining inflammation and tubular injury. Consistent with this concept, exogenous SPM administration attenuates albuminuria and inflammatory renal injury in experimental DKD, while strategies aimed at increasing omega-3 substrate availability to support endogenous pro-resolving pathways remain under active investigation and have yielded mixed clinical results to date. From a translational perspective, these observations position SPM pathways as mechanistic effectors linking omega-3 biology with renal protection in DKD and support their consideration as therapeutic targets and biomarkers of impaired resolution [93,145].

Beyond MaR1, other SPM classes have also demonstrated renoprotective actions in diverse experimental models of kidney injury. Notably, exogenous administration of LXA_4_ or its synthetic benzo-LXA_4_ analog consistently conferred protection in both chronic and metabolic settings. In a murine model of UUO, LXA_4_ treatment significantly attenuated interstitial fibrosis and collagen accumulation, while improving tubular morphology and preserving renal structure, supporting its potential as an early antifibrotic mediator [77]. Similarly, in obesity-related glomerulopathy, treatment with LXA_4_ or its stable benzo-LXA_4_ analog significantly improved renal outcomes in mice fed a high-fat diet. Lipoxin therapy reduced albuminuria, glomerular hypertrophy, mesangial matrix expansion, and oxidative stress, while attenuating tubulointerstitial collagen deposition and preserving overall renal architecture. Notably, these protective effects occurred without changes in renal macrophage counts, suggesting that the renoprotective actions of LXA_4_ were mediated indirectly through the modulation of systemic, adipose tissue-derived inflammation rather than direct local immune suppression [154].

Although clinical data on SPM modulation in CKD remain limited, early translational evidence supports their potential therapeutic relevance. In a randomized study including patients with stage 3–4 CKD, low-dose aspirin (100 mg/day) was shown to increase circulating concentrations of 15-epi-lipoxin A_4_, the aspirin-triggered epimer of LXA_4_, particularly in diabetic individuals. Baseline 15-epi-LXA_4_ levels were markedly lower in diabetic CKD compared to non-diabetic patients, but twelve months of aspirin therapy restored them to near-normal levels. This increase reached statistical significance (*p* = 0.017). These findings suggest that aspirin may partly exert its beneficial vascular and anti-inflammatory effects via the activation of COX-2-derived pro-resolving pathways, providing indirect evidence for pharmacologic enhancement of endogenous SPM signaling [155].

Similar renoprotective effects have been demonstrated for other SPMs. RvD1 protected against both ischemia/reperfusion- and LPS-induced AKI, reducing tubular injury, oxidative stress, and inflammatory cytokine release, partly through activation of regulatory T cells via the ALX/FPR2 receptor pathway [18,84]. Its aspirin-triggered analog (AT-RvD1) further alleviated proteinuria, collagen deposition, and tubulointerstitial fibrosis in post-septic and albumin-overload models by inhibiting TGF-β-dependent Smad2/3 signaling [156]. PD1 has shown comparable protective actions. In murine models of renal ischemia/reperfusion injury, PD1 administration reduced serum creatinine, interstitial fibrosis, and leukocyte infiltration, reflecting its anti-inflammatory and pro-resolving properties [98]. Moreover, PD1 activated the cytoprotective heme oxygenase-1 (HO-1) pathway in renal mesangial and tubular cells, enhancing antioxidant defenses and supporting tissue recovery [157].

**Figure 4:**
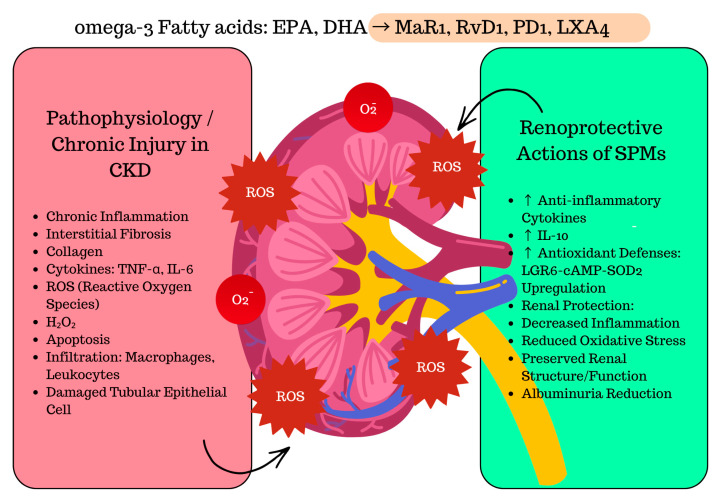
SPMs in CKD: Modulating Inflammation for Renal Protection. CKD—chronic kidney disease; H_2_O_2_—hydrogen peroxide; IL-6—interleukin-6; IL-10—interleukin-10; LGR6—leucine-rich repeat-containing G-protein-coupled receptor 6; MaR1—maresin 1; PD1—protectin D1; ROS—reactive oxygen species; RvD1—resolvin D1; TNF-α—tumor necrosis factor-alpha; EPA—eicosapentaenoic acid; DHA—docosahexaenoic acid; LXA4—lipoxin A4.

Collectively, these findings demonstrate that SPMs exert nephroprotective actions across multiple models of kidney injury by simultaneously modulating inflammation, oxidative stress, and fibrotic remodeling, thereby positioning them as promising candidates for future resolution-based therapies in CKD as it is shown on the Figure 4.

### 6.2. Safety, Pharmacological Interactions, and Co-Administration Considerations in CKD Therapy

#### 6.2.1. Cardiovascular Disease and Antithrombotic Therapy

Although SPMs are endogenously produced, their pharmacologic modulation, most achieved through omega-3 fatty acid supplementation, may interact with cardiovascular and nephroprotective agents frequently used in CKD. Concerns have been raised about a possible increase in bleeding risk associated with PUFAs, particularly in patients receiving concomitant antiplatelet or anticoagulant therapy. However, a large meta-analysis of 11 randomized trials including over 120,000 participants found no significant increase in overall, intracranial, gastrointestinal, or hemorrhagic stroke events in patients receiving omega-3 PUFAs compared with controls (*p* = 0.34). High-dose purified EPA modestly increased bleeding risk (absolute difference of 0.6%) without excess serious or fatal events. Meta-regression confirmed a dose-dependent association, while concomitant antiplatelet therapy (aspirin or clopidogrel) did not elevate bleeding risk [158].

In anticoagulated populations, observational data indicate no clinically relevant interaction between omega-3 supplementation and warfarin. In a retrospective cohort of 573 patients receiving long-term warfarin therapy for atrial fibrillation or venous thrombosis, concomitant use of fish or krill oil did not alter time in therapeutic range, bleeding incidence, or thromboembolic events compared with controls [159]. Perioperative RCT data likewise do not show increased surgical bleeding with fish-oil (EPA + DHA) supplementation and even report lower transfusion requirements compared with placebo [160].

#### 6.2.2. Lipid-Lowering Therapy and NSAIDs/COX-2 Considerations

PUFAs, particularly EPA and DHA, complement statin therapy by lowering triglycerides and remnant cholesterol fractions without significant pharmacokinetic interference. In a recent phase I study, co-administration of atorvastatin (40 mg) and omega-3-acid ethyl esters (4 g/day; EPA + DHA) over 14 days produced no clinically relevant pharmacokinetic interaction or increase in adverse events [161]. NSAIDs and COX-2 inhibitors may impair endogenous SPM biosynthesis and delay inflammation resolution. Experimental data indicate that COX-2 inhibition reduces the formation of omega-3-derived intermediates such as 18-hydroxyeicosapentaenoic acid (18-HEPE), thereby limiting downstream synthesis of E-series resolvins and other pro-resolving mediators. Similarly, LOX inhibition prolongs the resolution interval by suppressing SPM production. In contrast, statins enhance SPM generation through COX-2 S-nitrosylation, whereas short-term glucocorticoid therapy upregulates annexin A1 expression and promotes pro-resolving signaling. Therefore, in the context of SPM- or omega-3-based interventions, co-administration of resolution-supportive agents such as statins or short-course glucocorticoids, rather than chronic COX-2 or NSAID therapy, may help preserve the integrity of endogenous resolution pathways [61].

#### 6.2.3. Diabetes

A systematic review and meta-analysis of 30 randomized trials evaluated the tolerability of omega-3 supplementation (primarily EPA and DHA) in populations with type 2 diabetes, gestational diabetes, and related metabolic conditions. Across the included RCTs, intervention durations ranged from 4 to 72 weeks, while daily omega-3 doses varied widely (520 mg to 17.6 g). Overall, omega-3 supplementation was well tolerated at commonly used intake levels. Adverse effects were reported in only one trial using a dose of 17.6 g/day, exceeding commonly cited upper intake thresholds. By contrast, trials administering omega-3 at doses ≤5 g/day, including those with longer intervention periods, generally did not report adverse effects, although risk-of-bias assessments indicated variable methodological quality across studies [162].

In adults with type 2 diabetes, marine omega-3 supplementation (fish oil 1 g/day; 465 mg EPA + 375 mg DHA) was associated with adverse event rates similar to placebo in the VITAL-DKD randomized ancillary clinical trial (*n* = 1312; 5-year follow-up). Omega-3 and vitamin D_3_ were assigned in a 2 × 2 factorial design, and no significant interaction between the two interventions was observed. Omega-3 supplementation did not significantly affect change in eGFR and was not associated with significant differences in albuminuria measures versus placebo. Gastrointestinal bleeding was reported numerically more often with omega-3 than with placebo (28 vs. 17 events). Although bleeding was not a prespecified efficacy outcome and the trial was not powered for bleeding endpoints, this numerical difference may warrant clinical vigilance in patients with additional bleeding risk or concomitant antithrombotic therapy [163].

#### 6.2.4. Elderly and Pediatric Populations

In elderly patients, safety data for moderate-dose EPA/DHA supplementation are supported by the OMEMI randomized, placebo-controlled trial in patients aged 70–82 years with recent acute myocardial infarction. Daily supplementation with 1.8 g marine n-3 PUFA on top of contemporary secondary prevention, including widespread use of antiplatelet therapy and some anticoagulation, was not associated with an increased risk of major bleeding (BARC ≥ 2) over 24 months compared with placebo (*p* = 0.87). Although a subset of participants had impaired renal function, the trial was not designed for CKD-stratified safety analyses; therefore, conclusions specific to elderly patients with advanced CKD should be interpreted cautiously [164].

In pediatric populations, tolerability evidence is based primarily on RCTs in children and adolescents (6–18 years). A systematic review and meta-analysis of 31 trials (*n* = 1755) evaluating PUFA supplementation (omega-3 [EPA and/or DHA]-, omega-6-, or combined omega-3/omega-6 formulations) found no increase in adverse events compared with placebo, including gastrointestinal symptoms, although the certainty of evidence was low to very low and intervention durations were relatively short (8 weeks to 12 months) [165]. Limited randomized data are available in pediatric patients with advanced CKD. In a randomized, double-blind, placebo-controlled trial in children and adolescents (6–18 years) with ESRD receiving maintenance hemodialysis, once-daily omega-3 supplementation for 6 months (reported in the study as 1 g/day; the omega-3 syrup contained EPA 825 mg and DHA 550 mg per 5 mL dose) was reported to be well tolerated, with no life-threatening adverse events or non-compliance issues. Reported adverse effects were predominantly mild gastrointestinal symptoms, including flatulence (22.2%), fishy burping (18.5%), and diarrhea (7.4%). Interpretation is limited by the single-center design and modest sample size (with dropouts due to transplantation or death in both arms), underscoring the need for larger pediatric ESRD safety studies [117].

Overall, evidence from randomized trials and meta-analyses suggests a generally favorable safety and tolerability profile of prescription omega-3 fatty acids, although mild gastrointestinal and other adverse effects may occur; however, evidence on bleeding risk and drug–drug interactions (e.g., with antithrombotic therapy), particularly in CKD populations, remains limited [163,166].

### 6.3. Antioxidant Strategies and Inflammation Resolution in CKD

Patients with CKD, especially individuals on dialysis, are susceptible to vitamin deficiency because intake is often reduced and less varied, while anorexia or protein energy wasting and treatment associated losses further contribute. Since deficiency related symptoms are frequently mild or nonspecific, clinical testing is commonly restricted to a small number of routinely measured vitamins, most often folate, vitamin B12, and 25 hydroxyvitamin D. Available randomized data have not shown a reproducible improvement in kidney outcomes, cardiovascular endpoints, or patient centered outcomes with routine vitamin supplementation in CKD. Decisions about supplementation should therefore be made on an individual basis, taking into account dietary intake and the likelihood of dialysis associated losses, and nutritional vitamin D should be corrected using a tailored approach guided by baseline status and clinical context. Although vitamin E has been explored as an antioxidant strategy in CKD, evidence for clear clinical benefit remains inconsistent, and routine supplementation with vitamins A and E is generally not recommended because of limited efficacy and potential toxicity risk [167].

In addition to classical antioxidant supplementation, increasing attention has been directed toward redox-sensitive signaling pathways that actively promote oxidative stress in CKD. Among these, Na^+^/K^+^-ATPase has been recognized not only as an ion transporter but also as a signaling scaffold that enhances ROS generation through Src kinase-dependent mechanisms. Experimental studies in preclinical models relevant to CKD suggest that inhibition of Na^+^/K^+^-ATPase associated signaling can attenuate systemic oxidative stress and inflammatory cytokine signaling and may reduce fibrotic remodeling in preclinical settings, supporting its relevance as a non-classical antioxidant target in CKD [168].

Within this broader therapeutic context, antioxidant strategies targeting ROS-amplifying signaling pathways may conceptually complement resolution-focused approaches based on omega-3 fatty acids and SPMs. Unlike classical antioxidants, SPMs actively promote the termination of inflammation and restoration of tissue homeostasis rather than serving as classical, direct ROS-scavenging antioxidants [169]. Signaling mediated by the Na^+^/K^+^-ATPase oxidant amplification loop has been implicated in promoting ROS generation and inflammatory signaling in multiple cellular and animal models of oxidative stress-related injury, suggesting that modulation of this pathway may attenuate oxidative stress and its downstream pathophysiological effects in preclinical settings [168].

## 7. Challenges and Future Directions

The development and clinical translation of SPMs and omega-3 fatty acid derivatives in managing CKD face several critical challenges.

### 7.1. Translational and Pharmacological Barriers

A major obstacle in translating SPM-based therapies is their rapid metabolism and short half-life in vivo, largely attributable to enzymatic degradation by 15-prostaglandin dehydrogenase and other oxidoreductases. This rapid clearance limits effective bioavailability and therapeutic durability. Additionally, variability in the expression and activity of enzymes involved in SPM biosynthesis (such as ALOX5, ALOX12, and ALOX15) and genetic polymorphisms contribute to heterogeneous patient responses. To improve stability and potency, synthetic SPM analogs with increased resistance to metabolic degradation have been developed; however, balancing their anti-inflammatory effects without hampering essential immune defense functions remains a concern [170,171].

Implementing SPMs as reliable biomarkers for CKD is impeded by the lack of standardized, validated measurement techniques. Current methods, including liquid chromatography-tandem mass spectrometry (LC-MS/MS) and ELISA, vary in sensitivity, specificity, and reproducibility across laboratories, complicating data comparison and clinical interpretation. Large-scale multi-center validation studies are necessary to establish universal protocols, reference ranges, and clinically meaningful thresholds. Such efforts will facilitate the incorporation of SPM measurements into diagnostic and prognostic frameworks for CKD [140,172,173].

### 7.2. Need for Large-Scale Randomized Clinical Trials

While preclinical studies and small trials indicate potential benefits of omega-3 derivatives and SPMs in CKD, evidence from large, well-designed RCTs is limited. Many existing studies have insufficient power, short follow-up durations, lack of standardized dosing, and challenges with patient adherence, leading to inconclusive clinical outcomes. Robust RCTs involving diverse CKD populations, longer-term monitoring, and standardized endpoints are essential to confirm efficacy, optimize therapeutic regimens, and define safety profiles. These trials should also incorporate biomarker-guided patient stratification to identify responders and refine personalized treatment approaches [163,174,175].

### 7.3. Personalized Medicine and Patient Stratification

The heterogeneity in CKD pathophysiology and progression underscores the importance of personalized medicine. Multi-omics approaches combining genomic, proteomic, and metabolomic data can help stratify patients based on inflammatory status, omega-3 fatty acid metabolism, and other individual biological characteristics. Such stratification can enhance the precision of SPM-based interventions by tailoring therapies to patient-specific disease mechanisms. Additionally, addressing barriers to dietary adherence-such as socioeconomic factors, patient education, and taste preferences-is crucial for effective omega-3 supplementation. Healthcare provider involvement, especially nephrology-trained dietitians, can greatly improve patient compliance and nutritional outcomes [175,176,177].

Nanotechnology and targeted drug delivery systems represent promising strategies to overcome pharmacokinetic limitations of SPMs. Nanoparticle formulations, including biomimetic high-density lipoprotein (bHDL) nanoparticles targeting kidney injury markers, show improved targeting and sustained release in preclinical CKD models. These systems enhance drug accumulation at sites of renal injury, reducing systemic exposure and potential side effects. However, challenges remain regarding nanoparticle stability, manufacturing reproducibility, and regulatory approval. The successful clinical translation of nanomedicine in CKD will depend on optimizing delivery platforms to ensure safety and effectiveness [178,179,180].

The cost-effectiveness of SPM-based therapies is a pivotal factor shaping their clinical adoption. While omega-3 supplements are relatively affordable, synthetic SPM analogs and nanomedicine formulations may incur substantial development and production costs. Comprehensive economic analyses considering quality-adjusted life years (QALYs), healthcare utilization, and societal impact are needed. Demonstrating that these therapies can delay CKD progression, reduce hospitalizations, and improve survival will underpin reimbursement decisions and guideline endorsements [181].

### 7.4. Future Research Directions

Future studies should focus on elucidating the molecular mechanisms and temporal dynamics of SPM production and function during CKD progression. Combination therapies integrating SPMs with existing anti-inflammatory or anti-fibrotic agents, as well as standard CKD treatments (e.g., renin-angiotensin system blockers), warrant exploration. The development of point-of-care SPM biomarker assays can enable real-time therapeutic monitoring and adaptive treatment. Early engagement with regulatory agencies will be critical to navigate classification, safety requirements, and approval processes for these novel therapeutics [170,182].

## 8. Summary

CKD is driven by persistent low-grade inflammation, oxidative stress, and metabolic dysfunction that promote tubular injury, interstitial fibrosis, and cardiovascular complications. Key mechanisms include dysregulated innate immunity, activation of TLR/NLRP3 pathways, mitochondrial damage, and lipid metabolism disturbances leading to lipotoxicity.

SPMs including lipoxins, resolvins, protectins, and maresins are lipid mediators derived from omega-3 and omega-6 fatty acids that actively terminate inflammation without causing immunosuppression. Through receptors such as ALX/FPR2, ChemR23, GPR32, GPR18, and LGR6, they inhibit neutrophil infiltration, enhance efferocytosis, shift macrophages toward the M2 phenotype, limit oxidative stress, and counteract profibrotic signaling.

Omega-3 fatty acids (EPA, DHA) support these pathways and modulate lipid and inflammatory metabolism, although clinical results in CKD remain mixed. SPMs and omega-3 derivatives also show promise as biomarkers of inflammatory activity, CKD progression, and treatment response, but their clinical use is limited by analytical instability and lack of standardized assays.

Experimental models consistently demonstrate strong nephroprotective and antifibrotic effects of SPMs across ischemic, diabetic, septic, and obstructive kidney injury. Early clinical data suggest that enhancing endogenous resolution pathways is feasible, highlighting SPMs as promising targets for future CKD diagnostics and therapies.

## Figures and Tables

**Table 1:** Major classes of SPMs, their precursors, key biosynthetic enzymes, receptors, and selected renal effects.

Class of SPMs	Examples	Precursor	Key Biosynthetic Enzymes	Target Receptors	Selected Renal Effects	Reference
**LX (Lipoxins)**	LXA4, LXB4	AA (ω-6)	15-LOX, 5-LOX	ALX/FPR2	**UUO:** ↓ collagen, ↓ TNF-α/IFN-γ, ↓ NF-κB, MAPK/Akt/Smad, ↑ IL-10; anti-fibrotic and anti-inflammatory effects	[77]
**RvE (E-series resolvins)**	RvE1, RvE2	EPA (ω-3)	5-LOX, COX-2	ChemR23 (agonist), BLT1 (antagonist)	**UUO:** RvE1 → ChemR23 → PDGF-BB signaling → ↓ α-SMA/collagen; ↓ fibroblast proliferation	[87]
**RvD (D-series resolvins)**	RvD1, RvD2	DHA (ω-3)	15-LOX, 5-LOX	ALX/FPR2, GPR32 (RvD1, RvD3, RvD5), GPR18 (RvD2)	**I/R:** ↓ tubular injury, ↑ Treg, ↓ IFN-γ/TNF-α/IL-6; podocyte protection **AKI:** RvD1 → ALX/FPR2, GPR32 → ↓ NF-κB activation; ↓ renal cell apoptosis	[84]
**MaR (Maresins)**	MaR1, MaR2	DHA (ω-3)	12-LOX	LGR6 (GPCR), RORα	**I/R:** MaR1 → TLR4/MAPK/NF-κB → ↑ Nrf2 → ↓ TNF-α/IL-6, ↑ IL-10 → ↑ M2 **DKD:** MaR1 → LGR6 → ↑ cAMP/↑ SOD2 → ↓ ROS, ↓ inflammation, ↓ albuminuria, ↓ cytokines	[90,93]
**PD (Protectins)**	PD1, PDX	DHA (ω-3)	15-LOX	GPR37, PPARγ	**PD:** ↑ efferocytosis; macrophages → M2 (via PPARγ) → ↓ TNF-α/IL-6/MCP-1, ↑ IL-10; ↓ glomerulosclerosis	[97]

Abbreviations: AA—arachidonic acid; EPA—eicosapentaenoic acid; DHA—docosahexaenoic acid; 5-LOX—5-lipoxygenase; 12-LOX—12-lipoxygenase; 15-LOX—15-lipoxygenase; COX-2—cyclooxygenase-2; ALX/FPR2—lipoxin A_4_ receptor; BLT1—leukotriene B_4_ receptor 1; ChemR23—chemerin receptor 23; GPR18—G protein-coupled receptor 18; GPR32—G protein-coupled receptor 32; GPR37—G protein-coupled receptor 37; LGR6—leucine-rich repeat-containing G protein-coupled receptor 6; RORα—retinoic acid receptor-related orphan receptor-α; PPARγ—peroxisome proliferator-activated receptor-γ; PDGF-BB—platelet-derived growth factor-BB; α-SMA—alpha-smooth muscle actin; MAPK—mitogen-activated protein kinase; Akt—protein kinase B; Smad—SMAD signaling proteins; TNF-α—tumor necrosis factor-α; IL-6—interleukin-6; IL-10—interleukin-10; IFN-γ—interferon-γ; MCP-1—monocyte chemoattractant protein-1; TLR4—toll-like receptor 4; Nrf2—nuclear factor erythroid 2–related factor 2; UUO—unilateral ureteral obstruction; I/R—ischemia/reperfusion; Treg—regulatory T cell; ROS—reactive oxygen species; SOD2—superoxide dismutase 2; AKI—acute kidney injury; DKD—diabetic kidney disease; ↑—increase/upregulation; ↓—decrease/downregulation; →—activates/leads to/triggers.

**Table 2:** Specialized Pro-Resolving Mediators (SPMs) and their biomarker potential in CKD.

SPM (Specialized Pro-Resolving Mediator)	Detection Sample	Detection Limit	Source (Omega-3)	Main Actions in Inflammation	Cellular Mechanism/Effect	Potential Biomarker in CKD	Reference
**RvD1, RvD2 (D-series resolvins)**	Plasma, urine	~0.1–0.5 ng/mL (LC-MS/MS)	DHA	Inhibition of neutrophil chemotaxis	15-LOX, 5-LOX; suppression of NF-κB	Upregulation of Nrf2 and PPAR-γ	[125]
**RvE1, RvE2 (E-series resolvins)**	Plasma, urine	~0.2–0.8 ng/mL (LC-MS/MS)	EPA	Promotion of macrophage efferocytosis	Activation of M2 macrophages, reduction in ROS	RvE1/TNF-α ratio	[125]
**PD1, PDX (Protectins)**	Plasma, urine, renal tissue	~0.1–1 ng/mL (LC-MS/MS)	DHA	Endothelial barrier stabilization	Reduction in oxidative stress	Reduction in oxidative stress, PD1/CRP ratio	[126]
**MaR1, MaR2 (Maresins)**	Plasma, urine	~0.2–1 ng/mL (LC-MS/MS)	DHA	Limitation of tissue injury	Efferocytosis, modulation of NO/ROS signaling	Efferocytosis, modulation of NO/ROS signaling	[127]

Abbreviations: 15-LOX—15-Lipoxygenase; 5-LOX—5-Lipoxygenase; CRP—C-reactive protein; DHA—Docosahexaenoic acid; EPA—Eicosapentaenoic acid; LC–MS/MS—Liquid chromatography–tandem mass spectrometry; NF-κB—Nuclear factor kappa B; Nrf2—Nuclear factor erythroid 2–related factor 2; NO—Nitric oxide; PPAR-γ—Peroxisome proliferator-activated receptor gamma; PD1—Protectin D1; PDX—Protectin DX; RvD1—Resolvin D1; RvD2—Resolvin D2; RvE1—Resolvin E1; RvE2—Resolvin E2; ROS—Reactive oxygen species; SPM—Specialized pro-resolving mediator; TNF-α—Tumor necrosis factor alpha; MaR1—Maresin 1; MaR2—Maresin 2.

**Table 3:** Representative human studies of specialized pro-resolving mediators (SPMs) in chronic kidney disease (CKD) and diabetic kidney disease (DKD).

Study (Author, Year)	CKD Population	SPMs/Metabolites Measured	Sample & Method	Key Findings	Translational Stage	Reference
**Mas, E., 2016**	CKD (eGFR 15–60)	RvD1, 17-HDHA, 18-HEPE	Plasma, LC-MS/MS	Omega-3 supplementation increased SPM precursors and RvD1	Interventional feasibility	[137]
**Li et al., 2022**	T2DM vs. DKD	MaR1	Serum, ELISA	MaR1 significantly reduced in DKD; correlated with eGFR and UACR	Cross-sectional biomarker	[93]
**Bulu** **, A** **. et al., 2025**	DKD patients	MaR1	Serum, immunoassay	Lower MaR1 associated with albuminuria and inflammation	Disease severity marker	[142]
**Lidgard, B. et al., 2023.**	CKD stages 1–5	Multiple SPMs (RvD, MaR families)	Plasma/urine, LC-MS/MS	Progressive SPM depletion with CKD severity	Exploratory profiling	[143]

17-HDHA—17-Hydroxy docosahexaenoic acid; 18-HEPE—18-Hydroxy eicosapentaenoic acid; CKD—Chronic kidney disease; DKD—Diabetic kidney disease; eGFR—Estimated glomerular filtration rate; ELISA—Enzyme-linked immunosorbent assay; LC–MS/MS—Liquid chromatography–tandem mass spectrometry; MaR1—Maresin 1; RvD1—Resolvin D1.

## Data Availability

No new data were created or analyzed in this study.

## Abbreviations

The following abbreviations are used in this manuscript:

AA	Arachidonic Acid
ADR	Adriamycin
AKI	Acute Kidney Injury
ALA	Alpha-Linolenic Acid
ALOX5/12/15	Arachidonate 5-, 12-, and 15-Lipoxygenase
ALX/FPR2	Formyl Peptide Receptor 2 (lipoxin A4 receptor)
ANXA1	Annexin A1
AP-1	Activator Protein-1
apoC-III	Apolipoprotein C-III
AOPPs	Advanced Oxidation Protein Products
ARBs	Angiotensin Receptor Blockers
ASC	Apoptosis-Associated Speck-Like Protein Containing a CARD
AT-PD1	Aspirin-Triggered Protectin D1
AT-RvD1	Aspirin-Triggered Resolvin D1
ATLs	Aspirin-Triggered Lipoxins
α-SMA	Alpha-Smooth Muscle Actin
bHDL	Biomimetic High-Density Lipoprotein
BLT1	Leukotriene B_4_ Receptor 1
BUN	Blood Urea Nitrogen
cAMP	Cyclic Adenosine Monophosphate
ChemR23	Chemerin Receptor 23
CKD	Chronic Kidney Disease
COX-2	Cyclooxygenase-2
CRP	C-reactive protein
CTGF	Connective Tissue Growth Factor
CYP	Cytochrome P450 Monooxygenase
DAMPs	Danger-Associated Molecular Patterns
DCs	Dendritic Cells
DKD	Diabetic Kidney Disease
DHA	Docosahexaenoic Acid
DPA	Docosapentaenoic Acid
eGFR	Estimated Glomerular Filtration Rate
ELISA	Enzyme-Linked Immunosorbent Assay
EPA	Eicosapentaenoic Acid
ER	Endoplasmic Reticulum
ESKD	End-Stage Kidney Disease
ESRD	End-Stage Renal Disease
EGR-1	Early Growth Response-1
FAO	Fatty Acid Oxidation
FFAs	Free Fatty Acids
GPCRs	G Protein-Coupled Receptors
GPR18	G Protein-Coupled Receptor 18
GPR32	G Protein-Coupled Receptor 32
GPR37	G Protein-Coupled Receptor 37
HD	Hemodialysis
HDL	High-Density Lipoprotein
HDL-C	High-Density Lipoprotein Cholesterol
HO-1	Heme Oxygenase-1
HRMS	High-Resolution Mass Spectrometry
IDL	Intermediate-Density Lipoprotein
IFN-γ	Interferon-γ
IGF-1	Insulin-Like Growth Factor-1
IL-1β/6/10/13/18	Interleukin-1β/6/10/13/18
I/R	Ischemia/Reperfusion
IRI	Ischemia/Reperfusion Injury
JNK	c-Jun N-Terminal Kinase
LC	Long-Chain
LC–MS/MS	Liquid Chromatography–Tandem Mass Spectrometry
LGR6	Leucine-Rich Repeat-Containing G Protein-Coupled Receptor 6
LOX	Lipoxygenase
LPS	Lipopolysaccharide
LPL	Lipoprotein Lipase
LTA_4_	Leukotriene A_4_
LX	Lipoxins
LXA_4_	Lipoxin A4
LXRα	Liver X Receptor-α
MAPK	Mitogen-Activated Protein Kinase
MaR1	Maresin-1
MaR2	Maresin-2
MCP-1	Monocyte Chemoattractant Protein-1
MDA	Malondialdehyde
M1	Classically Activated (Pro-Inflammatory) Macrophage
M2	Alternatively Activated (Anti-Inflammatory) Macrophage
MUFAs	Monounsaturated Fatty Acids
NF-κB	Nuclear Factor Kappa B
NK cells	Natural Killer Cells
NO	Nitric Oxide
NOX2	NADPH Oxidase 2
NOX4	NADPH Oxidase 4
Nrf2	Nuclear Factor Erythroid 2–Related Factor 2
NSAIDs	Non-Steroidal Anti-Inflammatory Drugs
NLRP3	NOD-, LRR-, and Pyrin Domain-Containing Protein 3
OS	Oxidative Stress
PAMPs	Pathogen-Associated Molecular Patterns
PD1	Protectin D1
PDX	Protectin DX
PDGF-BB	Platelet-Derived Growth Factor-BB
PGC-1α	Peroxisome Proliferator-Activated Receptor-γ Coactivator-1α
PGH_2_	Prostaglandin H_2_
PPARα	Peroxisome Proliferator-Activated Receptor-α
PPARγ	Peroxisome Proliferator-Activated Receptor-γ
PRRs	Pattern Recognition Receptors
PUFAs	Polyunsaturated Fatty Acids
QALYs	Quality-Adjusted Life Years
RAAS	Renin–Angiotensin–Aldosterone System
RCT	Randomized Controlled Trial
RNS	Reactive Nitrogen Species
RORα	Retinoic Acid Receptor-Related Orphan Receptor-α
ROS	Reactive Oxygen Species
RvD1	Resolvin D1
RvD2	Resolvin D2
RvE1	Resolvin E1
RvE2	Resolvin E2
SDA	Stearidonic Acid
sEH	Soluble Epoxide Hydrolase
SFAs	Saturated Fatty Acids
SOD	Superoxide Dismutase
SOD2	Superoxide Dismutase 2
SPMs	Specialized Pro-Resolving Mediators
SREBP-1	Sterol Regulatory Element-Binding Protein-1
SREBP-1c	Sterol Regulatory Element-Binding Protein-1c
TEC/TECs	Tubular Epithelial Cell / Tubular Epithelial Cells
TG	Triglycerides
TGF-β	Transforming Growth Factor-β
TGF-β1	Transforming Growth Factor-β1
TLR4	Toll-Like Receptor 4
TLRs	Toll-Like Receptors
TNF-α	Tumor Necrosis Factor-α
Treg	Regulatory T Cell
TRLs	Triglyceride-Rich Lipoproteins
TXA_2_	Thromboxane A_2_
UHPLC	Ultra-High-Performance Liquid Chromatography
UUO	Unilateral Ureteral Obstruction
VLDL	Very-Low-Density Lipoprotein
Wnt	Wingless/Int-1 Signaling Pathway
